# A Major Update and Improved Validation Functionality in the mwtab Python Library and the Metabolomics Workbench File Status Website

**DOI:** 10.3390/metabo16010076

**Published:** 2026-01-15

**Authors:** P. Travis Thompson, Hunter N. B. Moseley

**Affiliations:** 1 Markey Cancer Center, University of Kentucky, Lexington, KY 40506, USA; ptth222@uky.edu; 2 Superfund Research Center, University of Kentucky, Lexington, KY 40506, USA; 3 Department of Molecular and Cellular Biochemistry, University of Kentucky, Lexington, KY 40506, USA; 4 Institute for Biomedical Informatics, University of Kentucky, Lexington, KY 40506, USA

**Keywords:** metabolomics workbench, data validation, data deposition, Python package

## Abstract

**Background:** The Metabolomics Workbench (MW) is a public scientific data repository consisting of experimental data and metadata from metabolomics studies collected with mass spectroscopy (MS) and nuclear magnetic resonance (NMR) analyses. Although not as rapidly as in the past, MW has steadily evolved, updating its mwTab and JSON deposition text file formats and its web-based infrastructure. However, the growth of MW has been exponential since its inception in 2013 and continues to be exponential, with the number of datasets hosted on the repository increasing by 50% since April 2024. As part of regular maintenance to keep up with changes to the mwTab file format and an earnest effort to use MW datasets in meta-analyses, the mwtab Python package has been updated. **Methods:** Updates include better error handling for batch processing, better parsing to read more files without error, and extensive improvements to the validation capabilities of the package. These updates also required our mwFileStatusWebsite to be updated and improved. **Results:** We used the enhanced validation features of the mwtab package to evaluate all available datasets in MW to facilitate improved curation, FAIRness of the repository, and reuse for meta-analyses. **Conclusions:** Version 2.0.0 of the mwtab Python package is now officially released and freely available on GitHub and the Python Package Index (PyPI) under a Clear Berkeley Software Distribution (BSD) license, with documentation available on GitHub. The updated mwFileStatusWebsite is also officially in its 2.0.0 version.

## 1. Introduction

The National Institutes of Health (NIH) Reform Act of 2006 established the NIH Common Fund to support cross-cutting, trans-NIH programs. The Common Fund’s Metabolomics Program, started in 2012 and supported until 2022, established the Metabolomics Common Fund’s National Metabolomics Data Repository, known as the Metabolomics Workbench (MW), as a longstanding, public repository for national and international metabolomics data and metadata [1]. This repository consists of experimental data and related metadata for metabolomics studies collected with mass spectroscopy (MS) and nuclear magnetic resonance (NMR) analytical platforms. MW is organized into specific projects, studies, and analyses (experiments). Each analysis can be accessed and downloaded in the mwTab tabular format [1] or as JavaScript Object Notation (JSON) formatted files [2,3], both of which are text-based. MW offers a web interface to search, access, deposit, or download organized metabolomics data and metadata. Additionally, MW offers a REpresentational State Transfer (REST) interface to download and view (meta)data [4]. Since MW’s establishment in 2013, the repository has grown exponentially, roughly doubling every two to three years. As of 22 October 2025, MW contained a total of 2466 projects, 3795 studies, and 6125 analyses.

A few software packages, protocols, and databases have been developed over the years to facilitate access and utilization of MW and its datasets. A protocol for using MW datasets was developed for MetaboAnalysis 5.0 [5]. The Reference Set of Metabolite Names (RefMet) and associated metabolite classification system facilitate meta-analyses across MW datasets [6]. The Metadata from the Experimental SpreadSheets Extraction System (MESSES) facilitates the generation of mwTab-formatted deposition files [7]. MetabolomeXchange aggregates data from MW and other metabolomics repositories [8]. The mwtab Python package, originally released in 2017, facilitates Python programmatic access to mwTab-formatted files from MW [9].

The mwtab package also has basic facilities for creating mwTab-formatted files to facilitate more automated deposition processes, which were greatly enhanced by the MESSES package. We then released the first major version, 1.0.1, written in Python 3, which dramatically increased the functionality of the mwtab package [10]. Release 1.0.1 included updates to reflect the evolving changes in the mwTab format specification, incorporated methods that utilized the MW’s REST interface, and provided a range of new validation functionality for quality control and curation purposes. However, the overall goal of the mwtab Python package is to promote the FAIR data principles of findability, accessibility, interoperability, and reusability [11,12] with respect to MW. We are now releasing the next major version, 2.0.0, which contains updates for every module in the package and adds two new modules. A summary of the changes to each module is shown in Table 1.

**Figure 1:**
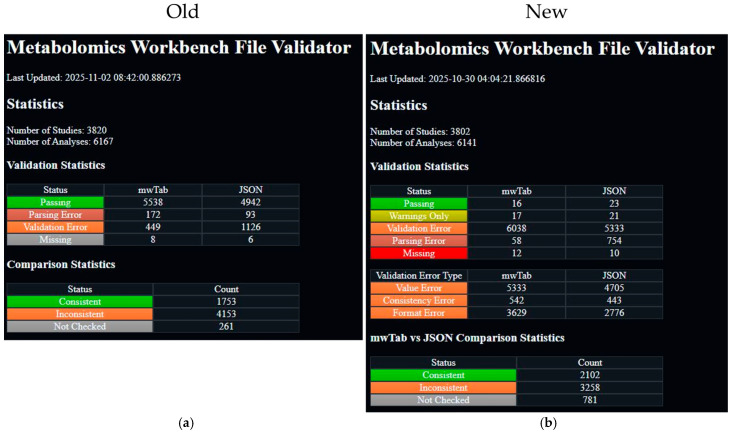
Screenshots from the previous and new versions of the mwFileStatusWebsite: (**a**) screenshot of the previous version of the mwFileStatusWebsite; (**b**) screenshot of the current version of the mwFileStatusWebsite. Not shown is the list of studies and their analysis IDs below these summary statistics, where the analysis ID is colored to match its validation status. For example, if the analysis passed validation, then the analysis ID would be inside a green box, just like “Passing” is in the “Status” column.

The most significant changes in the new mwtab release are in the validations. Specifically, many more validations have been added, and they have been classified based on the nature of the issue with the mwTab file. These changes necessitated an update to another related package and website that we maintain, the mwFileStatusWebsite [13]. This package is also being released to a new 2.0.0 version. The most significant changes to the website are adding a new status, “Warnings Only” with a yellowish-green color, and adding a new “Validation Error Type” table that breaks down the validation errors into the new classifications and links to new pages that filter the files based on those classifications. Also, the color range was extended to include a bright red color for “Missing” instead of the previous gray color. Also, the order of statuses was slightly changed, with “Parsing Error” put after “Validation Error”, since parsing issues are generally considered more severe than validation issues. These changes are best illustrated in Figure 1.

## 2. Materials and Methods

### 2.1. Updates to the mwTab Format

The mwTab format specification was last updated on 1 August 2023. The updated mwTab format specification is available on the MW website [14].

The mwTab format has remained mostly the same except for a few additions. These additions to the mwTab format include (1) changing 5 attributes in the CHROMATOGRAHPY section from optional to required, (2) deprecating the NMR_BINNED_DATA section in favor of putting that data in a separate file indicated by a NMR(MS)_RESULTS_FILE attribute in the NM(MS) section, and (3) similarly allowing and preferring unassigned mass spec measurements to go in a separate file indicated by the MS_RESULTS_FILE attribute in the MS section. Many of these items are not explicitly stated as being updates to the mwTab format, but as of November 2025, are present in a significant number of analyses. Tracking updates to the mwTab specification is not easy for 3 reasons. First, older versions of the specification are not readily available from the MW website, only the most recent one. Second, although there is a VERSION attribute in the mwTab file header, it is not effectively used. All analyses just have the value of ‘1’ for this attribute, meaning that the version of the specification used to generate the file is unknown. Third, not all changes are explicitly stated in the specification file and instead must be inferred through observation of the files or use of the online submission system. When using the web forms to try to submit to MW, there are differences in what the forms require vs. what is stated in the mwTab specification.

### 2.2. mwtab Package Implementation

The mwtab Python package previously consisted of nine modules: mwtab.py, tokenizer.py, fileio.py, converter.py, mwschema.py, validator.py, mwextract.py, mwrest.py, and cli.py. In the 2.0.0 release, changes were made to all of those modules, and two additional modules, metadata_column_matching.py and duplicates_dict.py, were added to the package. The updates to the package modules largely fall into three main categories: (1) updates to improve error handling when operating on multiple files, (2) updates to improve the main functionality of the module or fix bugs, and (3) a great expansion of the mwTab format and metadata validation capabilities, along with the addition of some data validation capabilities.

The cli.py, converter.py, fileio.py, mwextract.py, and mwrest.py modules were all upgraded with code to recover from an unexpected error when processing one file out of multiple files. Previously, for some functions, if an unexpected error was encountered while processing one file in a batch of files, the overall batch process would error, and the remaining files would not be processed. Many of the aforementioned unexpected errors were also investigated, and the code was improved to handle those situations. For example, if an output path was specified, but one or more of the directories did not exist, this would cause an error. Code was added to check for this situation and create the directories that do not exist. The tokenizer.py module also underwent this improvement process, but did not need the upgrade to recover when batch processing, because it does not do batch processing.

The mwtab.py module received significant upgrades and additional functionality. The most significant improvement is the ability to handle duplicate keys. Round-trip testing refers to using the package to read in a file and then write it back out with no information loss. When performing this testing, it was discovered that there was information loss for some files, and it was due to some parts of the file having duplicate names. For example, if two columns in the METABOLITE_DATA or METABOLITE sections have the same name, then only the last column with that name would be read in. This is due to Python dictionaries not being allowed to have a key that points to two different values, so when the second column was added to the same dictionary that the first was already in, it overwrote the first column’s data. This was a difficult error to find and fix. Difficult to find because there is no warning about this duplicate key from Python’s built-in json package, used to read in the JSON version of mwTab files. Difficult to fix because although duplicate keys are technically allowed in JSON, they are highly discouraged, and therefore, such a rare occurrence that there is no standard way in Python to read and write them out. The new module, duplicates_dict.py, was created to handle this situation. It contains a single class, DuplicatesDict, that is used instead of the regular Python dictionary to be able to read in and write out duplicate keys in JSON. Other improvements to the mwtab.py module include working hand-in-hand with the tokenizer.py module to improve parsing, adding convenience properties and methods when working with the API, and improving writing out files by setting the section/key order to a predefined order so all files write out with the same section/key order.

The validator.py and mwschema.py modules work together and received the most significant changes. The mwschema.py module was completely rewritten using the jsonschema Python package [15,16] instead of the schema Python package. This was to enable both better error messaging and better validation. The jsonschema package has better support in both of these areas. The schemas were also improved to validate the values of some attributes with a more constrained value space, such as STUDY_ID. Several functions were also created to help construct the regular expressions used for value validation and to help construct the custom error messages for those attributes. The validator.py module uses the schemas in the mwschema.py module for the validations that are easily handled by jsonschema. So, improvements in the mwschema.py module translate to improvements in the validator.py module. In addition, the validator.py module received substantial improvements outside of the improvements in the mwschema.py module. A host of consistency validations were added, for example, validating that the subject sample factors in the SUBJECT_SAMPLE_FACTORS section and the METABOLITE_DATA section are the same. Many validations were added to detect duplicates, such as duplicate sample names, duplicate metabolite names, and duplicate rows in tables.

The most extensive validation, in terms of development time, was significantly improving and extending the validation on columns in the METABOLITES table. Previously, there was a validation that looked for column names that looked like certain standard column names and printed a message to the user to change them to the standard name. For example, “retention_time” would be a standard column name, but you can find variations on that name, such as “ret time”, “ret_time”, or “retention time”. This validation was greatly expanded. Originally, 11 standard column names were validated, and that has now increased to 56. The method used to identify these column names was also improved, and an iterative test-improvement loop using all of the column names in the MW repository was performed to fine-tune the method for each name to reduce false positives and false negatives. Additionally, the validation was extended to also validate the values of each of these columns, not just the name. A similar test-improvement loop was used to fine-tune the value validation. These extensive improvements spawned the new module, metadata_column_matching.py. This new module contains all of the new classes, functions, and resulting class objects used by validator.py to perform the standard column name and value validation. Since the code in the metadata_column_matching.py module could be used to validate the names and values of any kind of data in tabular form, we added extensive documentation (https://moseleybioinformaticslab.github.io/mwtab/metadata_column_matching.html, accessed on 13 December 2025) on how to use this module outside of the mwtab package for that purpose.

The validation messaging also received an overhaul. Internally, the validation is performed on the internal representation of the mwTab file, and validation messages reflect that in the previous version. This caused the messages to make a bit less sense when referring to mwTab files in the tabular form, and also caused a lot of repeating spurious messages about what was indeed 1 error. Now, validation is aware of whether it is validating a tabular or JSON version and crafts messages specific to the file type. The spurious repeating messages have also been condensed to just 1 message. The last noteworthy change to validation is that it no longer just prints messages. It now also returns a list of dictionaries, where each dictionary is a validation message along with tags categorizing the message, a unique ID, a short name, and keys for the section and sub-section that generated the message. This change was largely motivated by the changes made to the mwFileStatusWebsite, which made it much easier to improve the information provided by the website.

### 2.3. mwFileStatusWebsite Updates

Updates to the mwFileStatusWebsite were not quite as extensive as those to the mwtab package, but were required due to the extensive changes to the mwtab package. Since the website had to be updated due to the changes in the mwtab package, other maintenance or modernization updates were also performed at the same time. These updates included switching the installation method to using a pyproject.toml file [17] instead of the older setup.py file, switching to a src distribution package organization, and switching to versioning using GitHub tags and the setuptools-scm package [18]. Some other minor updates were made to the site functionality. Previously, when a user clicked on a specific study or analysis to see more detailed information, only one set of study or analysis details could be open at a time. We changed the cascading style sheets (CSS) so that any number of studies or analysis details can be displayed at the same time. The largest and most impactful changes came downstream from the changes to the mwtab package. Previously, when the mwtab package had many fewer validations, there were many more mwTab files that reported no validation issues and were put in the “passing” category. However, after adding many more validations to the mwtab package, most mwTab files have some sort of validation issue. In order to make the site a little more useful, we decided that it needed to include new categories and sub-categories of validation issues. To that end, we added a “Warnings Only” category, which is for mwTab files that only have warnings in the validation output, and we added the sub-categories of “Value Error”, “Consistency Error”, and “Format Error” to sub-categorize the validation errors. A “Value Error” is where the value of a subsection or column is incorrect. For example, putting ‘5 W’ for a subsection asking for a voltage. A “Consistency Error” is where the same values should be in 2 different locations, but they are not. For example, when there are metabolites in the METABOLITES table that are not in the DATA table. A “Format Error” is where the file does not follow the mwTab specification. For example, the file has sub-sections that do not exist or are missing required subsections. The desire to add these additional sub-categories to the website drove the changes to the mwtab package to support them, so the mwtab package changes drove changes to the website, and the website then drove changes to the mwtab package. Having the validation output in the previously described list of dictionaries was the best way to support these updates to the website. Previously, the website was parsing the text output from validation, but now it is much easier to utilize the list of dictionaries output to obtain the category and sub-category information needed by the website. Figure 1 illustrates the additional category and sub-categories, and the reader can visit the website at https://moseleybioinformaticslab.github.io/mwFileStatusWebsite/ (accessed on 13 December 2025) to see its functionality. We also took the time to add unit testing to the site code, such that over 99% of the code lines are now covered.

### 2.4. Evaluation of the Metabolomics Workbench Repository

The mwtab package functionality was evaluated on every available mwTab- and JSON-formatted file available from MW (as of 22 October 2025). A total of 6125 analysis IDs were available, and the downloading of both mwTab- and JSON-formatted data files was attempted through the MW REST interface.

The files from MW were evaluated for a number of formatting, value, and consistency errors/issues. First, the successful download of each analysis file through MW’s REST API was tested in both mwTab and JSON format. Second, the successful parsing of each downloaded file into MWTabFile objects was tested. Third, each parsed file was validated using the validation methods in the mwtab Python package. Fourth, the consistency between parsed data from mwTab and JSON formats was tested.

Downloading was performed only using the new version of the mwtab package, but parsing and validation steps were performed using both the previous version of the mwtab package and the new version. Consistency between the mwTab and JSON formats parsed by both the new and previous versions of the mwtab package was tested using the updated consistency checking code in the mwFileStatusWebsite.

## 3. Results

As of 22 October 2025, a total of 6125 analyses were available for download through MW’s REST interface. With the mwtab 2.0.0 version, we applied a series of evaluations on each analysis in both the mwTab and JSON formats. These evaluations were performed in a natural progression, starting with whether the analysis file could be downloaded and parsed, followed by whether the mwTab and JSON files were consistent with each other, and finally applying an extensive list of validation tests.

### 3.1. Analysis IDs with Files Missing from the Metabolomics Workbench

When we attempted to download all available analyses, a number of analyses were not present for download in a given format. Of those analyses, 8 could not be downloaded in mwTab format, and 6 could not be downloaded in JSON format. The specific analysis IDs are given in Table 2. Only blank pages were present for these files.

It can be seen by comparing the analysis IDs in Table 2 that 6 out of the 8 files are shared between both formats. Further investigation was performed by finding these analysis IDs on the Metabolomics Workbench website through the summary of all studies. The files available for download that way were not all blank like they are when using the REST interface, but when they are not blank, they are also not in the correct format. For example, the mwTab format file for AN002312 contains only the tables that would be in the DATA and METABOLITES sections. It is not clear why most of the analysis IDs that could not be downloaded overlap across formats, but not all of them. This seems to imply a lower-level issue specific to each analysis ID, since most are not available in either format.

### 3.2. Analysis Files Which Could Not Be Parsed

Of the 6117 downloaded analyses in mwTab format, 51 files could not be parsed into MWTabFile objects. Of the 6119 downloaded analyses in JSON format, 124 files could not be parsed into MWTabFile objects. For 37 analysis IDs, both the mwTab-formatted file and the JSON-formatted file could not be parsed.

Of the 51 mwTab files that could not be parsed, 30 files contained formatting errors in the additional data section of the SUBJECT_SAMPLE_FACTORS, where either the semicolon delimiter between key–value pairs was missing, or there was an additional ‘=’ character that should be separating the key and value. A total of 13 files had formatting errors, where there were lines without the capitalized sections at the beginning. This was usually due to the value of a section containing a value with a carriage return. Five files had formatting errors where they had a line that was missing the tab after the capitalized section at the beginning of the line. Two files had formatting errors where the factors had an additional ‘:’ character that should be separating the key and value. One file had a very badly formatted METABOLITES_DATA table that had multiple MS_METABOLITE_DATA_END lines between the rows of the table. There should only be one MS_METABOLITE_DATA_END line at the very end of the table.

Of the 124 JSON files that could not be parsed, 69 files were simply badly formatted JSON. For instance, if the mwTab-formatted version of the file had an extra ‘=’ character as described above, then that would translate into an extra invalid character in the JSON version. A total of 48 files had non-dictionary values in the tabular data. The METABOLITES_DATA and METABOLITES tables within the mwTab-formatted version become translated into a list of dictionaries in the JSON version, but for some files, one or more of these dictionaries instead have a non-dictionary value like “false”. When compared to the mwTab-formatted version of the same file, the non-dictionary values correspond to a row in a table, and the value for that row is not “false”. This means that the JSON version is missing data that is in the mwTab version. We believe that the JSON files are created by parsing the mwTab version of the file, and that for some reason, certain rows fail to parse correctly and result in the “false” value. By default, the mwtab package does not parse these files, but in version 2.0.0, there is a “force” CLI option and API function parameter that instead ignores these values and allows the files to be parsed. The last 7 files that could not be parsed are due to having duplicate keys at the highest level of the JSON, for instance, having 2 “MS” or “MS_METABOLITE_DATA” keys. Although one of the improvements in this release is being able to handle duplicate keys, that is only for values in lower levels of the data, such as Factors within the SUBJECT_SAMPLE_FACTORS. Duplicate keys at the highest level are much harder to deal with. Also, if one looks at the mwTab-formatted version of these 7 files, the duplicated keys in the JSON do not correspond to duplicate sections in the mwTab.

The mwTab package has a “convert” command that allows a user to convert between the mwTab-formatted version of a file and the JSON version of a file and vice versa. When we use this command to convert all of the downloaded mwTab-formatted files into JSON files, all of the converted JSON files are parsable. In other words, converting the mwTab-formatted files to JSON files fixes 100% of the JSON file parsing errors for the files that have a mwTab-formatted version without parsing errors.

For a full list of files with parsing errors, see Supplemental Table S1.

### 3.3. Comparing Parsability Between mwtab Version 1.2.5 and Version 2.0.0

When using the previous version of the mwtab package (1.2.5), 162 analysis files in mwTab format could not be parsed into MWTabFile objects, and 69 analysis files in JSON format could not be parsed into MWTabFile objects.

Of the 162 files in the mwTab format, 20 overlap with the 51 unparsable files when using the new 2.0.0 version. The 31 files that are parsable when using the previous version of the package have arguably a worse issue. Although the package can parse them, the resulting data would be wrong and would not match the file that was read in if the package were used to write out what it had parsed. The root cause is the aforementioned issue in the SUBJECT_SAMPLE_FACTORS, where some of the syntax characters can be missing, or there are extra ones due to the data uploader using those characters in the key or value. The previous version of the mwTab package used a more naïve approach to parsing the SUBJECT_SAMPLE_FACTORS that was unaware of this issue, resulting in incorrect MWTabFile object representations. Naïve approaches to parsing explain the other 142 unparsable files. Detailing every naïve assumption that causes an issue in parsing is not within the scope of this paper, but 112 come from the SUBJECT_SAMPLE_FACTORS and the other 30 come from tab placement issues throughout the sections.

Of the 69 files in the JSON format, all of them overlap with the 124 unparsable files when using the new 2.0.0 version. The 55 files that are parsable by the previous version and not the new version are the 48 files with non-dictionary table values and the 7 files that have duplicate keys at the top level. Even though the new version of the package results in fewer files being parsable, it is better overall because those issues in a file can cause many more pernicious issues deeper in a data workflow if they were parsed in with the more naïve method used in the previous version of the package. Also, 48 of the 55 files can be parsed in if the aforementioned “force” option is utilized.

We will also mention here that 10 of the files in the mwTab format and 24 of the files in the JSON format cannot be validated when using the previous version of the package because the package would crash. This is partly due to naïve coding assumptions and partly due to files from the Metabolomics Workbench not being as reliably formatted as you might like or expect. The issues causing the validation code to crash are due to certain assumptions that would be reasonable and would be expected of many data providers. For example, in a JSON object, data that is not present can be represented by having the key with a null value or by not including the key. A reasonable expectation is that a data provider would choose one of those methods and then use that as the standard across their datasets. They would not have some datasets that had keys with null values and some without the key. Unfortunately, datasets from the Metabolomics Workbench do have this issue. The new version of the mwtab package fixed all of these errors in the validation code and does not crash on any of the 6117 files in the mwTab format or any of the 6119 files in the JSON format.

For a full list of files with parsing errors and validation crashes, see Supplemental Tables S2 and S3.

### 3.4. Consistency Errors Between mwTab- and JSON-Formatted Files

**Figure 2:**
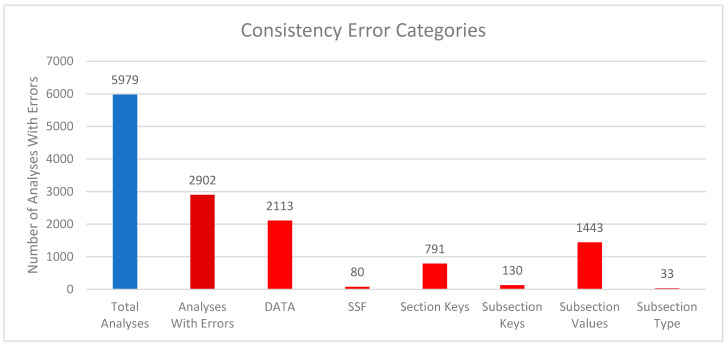
Column chart showing the breakdown of reasons for the inconsistencies between downloaded mwTab-formatted and JSON-formatted files for the new 2.0.0 version of the mwtab package. Blue represents the total number of analyses available from MW and parsable by the package. The deep red represents the number of analyses with consistency errors. The bright red represents which part of the analysis files had consistency issues.

**Figure 3:**
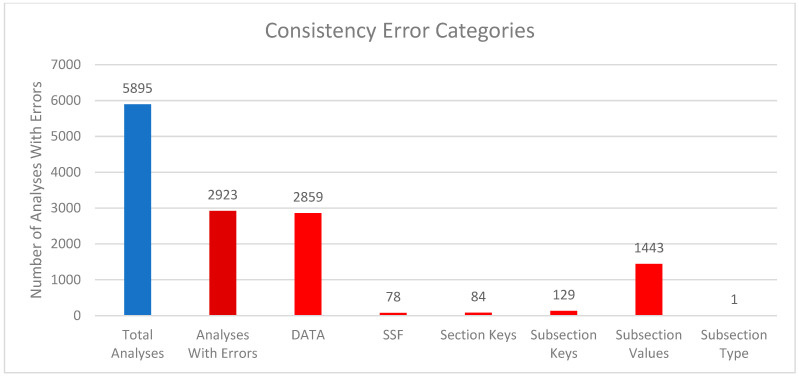
Column chart showing the breakdown of reasons for the inconsistencies between downloaded mwTab-formatted and JSON-formatted files for the previous 1.2.5 version of the mwtab package. Blue represents the total number of analyses available from MW and parsable by the package. The deep red represents the number of analyses with consistency errors. The bright red represents which part of the analysis files had consistency issues.

We compared the internal package representations when reading in the downloaded mwTab version of the analysis with the downloaded JSON version of the analysis to see how consistent they are with each other. In this context, “consistent” means that the MWTabFile objects contain the same data. We did this comparison for both the new 2.0.0 version of the mwtab package and the previous 1.2.5 version. For the new version of the 5979 parsable, non-missing files, 3077 were consistent with each other, and 2902 were inconsistent with each other. For the 1.2.5 version, of the 5895 parsable, non-missing files, 2944 were consistent with each other, and 2951 were inconsistent with each other. The reasons for the inconsistencies were investigated. Figure 2 and Figure 3 show a summary of the categorized reasons for inconsistency for the new and previous versions of the mwtab package, respectively.

The “DATA” category refers to the 2 formats having differences in the DATA or METABOLITES sections. Similarly, “SSF” refers to the 2 formats having differences in the SUBJECT_SAMPLE_FACTORS section. “Section Keys” means there were differences between the sections, for example, one may have a CHROMATOGRAPHY section, and one may not. Similarly, “Subsection Keys” means there were differences within a section, for example, one might have a RESULTS_FILE key in the MS section, and one may not. “Subsection Values” means that for the same subsection within a section, the values were not the same between the 2 formats. Lastly, “Subsection Type” is similar to “Subsection Values”, but not only is the value different, but the data type is different. For example, a subsection might have a string (text) value in one format, but a list of strings in the other.

Comparing Figure 2 and Figure 3 closely, there are 2 significant discrepancies between the new and previous versions of the mwtab package. The new version has significantly more “Section Keys” inconsistencies, and the previous version has significantly more “DATA” inconsistencies. Nearly all of the extra “Section Keys” inconsistencies come from 736 mwTab-formatted files that have a “#FACTORS” line in them. There is not supposed to be a FACTORS section at all, but for some reason, these files have a line like that in them. This causes the mwtab package to create an empty FACTORS section in the MWTabFile object when reading in that file, but the corresponding downloaded JSON version of the file does not have that section, thus the inconsistency. These do not show up in the previous version of the mwtab package because of a small difference in parsing. The previous version does not create the FACTORS section because it is empty, but this behavior can cause other issues, so it was changed in the new version. The extra “DATA” inconsistencies also come from a difference in parsing. The mwTab-formatted versions have tab-delimited data tables for the DATA and METABOLITES sections. There are 2 different kinds of tab issues that can happen with these tables. One is that there can be extra tabs with no data tacked on to the end of rows, and not necessarily every row, such that the table can look like it has several extra columns with no name and no values. The other tab issue that can happen is that when a row has a blank value for the last column(s), the tabs that are supposed to be there are not there. This makes it look like the row only has some of the columns. The previous version of the mwtab package parsed these tables more naively and relied on the number of tabs in each row being correct. The new version of the package is not so naïve and harmonizes the columns for each row, making it more consistent with the downloaded JSON.

We also used the mwtab package’s “convert” command through its CLI to convert the downloaded mwTab-formatted files into JSON-formatted files and compared them. Unsurprisingly, they are 100% consistent. This is another reason why we recommend working with our converted JSON files over those directly downloaded.

The mwFileStatusWebsite does this consistency check between the downloaded mwTab- and JSON-formatted files every week, along with its validation of the files, and was updated to fix some inaccuracies and address changes made in the new version of the mwtab package.

### 3.5. Validation Issues

The improvements in validation are the most extensive changes made in the new version of the mwtab package. Table 3 shows the number of analyses that fall into a few general validation issue categories when using the previous version of the mwtab package to validate.

Table 4 is similar to Table 3 but expands into more categories when using the new version of the mwtab package, and Table 5 gives more in-depth descriptions for each category.

The categories had to be expanded in Table 4 for the new version of the mwtab package, because “warnings” are a new concept in this version, as are the “consistency”, “value”, and “format” categories of validation issues. “Warnings Only” was added as a new category in the mwFileStatusWebsite, since many new validation warnings were introduced in the new version of the mwtab package. Warnings are less severe than an outright error and have a chance of being a false positive. We felt adding this category would add more gradations and nuance when evaluating files from MW. We also added categories to the validation issues: “consistency”, “value”, and “format”. The “consistency” category in this context refers to consistency between sections within the file. For example, if there is a Sample ID in the METABOLITE_DATA table that is not in the SUBJECT_SAMPLE_FACTORS section, then there is a consistency error between those 2 sections. A “value” error is when there is an issue with the value somewhere. For example, if there are 2 metabolites in the METABOLITES table with the same name, then that is a value error because one of the values is likely incorrect. Lastly, a “format” error is when there is an issue that violates the mwTab specification. For example, if the required sub-section SUBJECT_TYPE is not present in the SUBJECT section, that is a format error because the mwTab specification requires this field to be present. The prior versions of the mwtab package had primarily focused only on “format” errors.

The “Only New Errors”, “Only New Errors and Warnings”, and “Previous Standard Name Errors” categories were added to Table 4 to help show how so many files went from “Passing” in the previous version of the mwtab package to “Errors” in the new version. We can see from “Only New Errors” that around 2600 files are now not passing solely because of new validations introduced. We can see from “Only New Errors and Warnings” that around 5700 files are now not passing due to new validations or warnings. The gap between “Only New Errors” and “Only New Errors and Warnings” is explained with the “Previous Standard Name Errors” category, but some preamble is required to understand this category. The previous version of the mwtab package had a less sophisticated version of a validation that was greatly expanded in the new version of the package. This validation looks for certain standard columns in the METABOLITES table that do not have the corresponding standard name and prints a warning to the user to rename them. For example, one standard column name is “moverz_quant”, but this could appear in datasets as “mz”, “m/z”, “moverz”, etc. In the new version of the mwtab package, this validation is much more robust for the names that were covered in the previous version and covers many more names. This validation in the new version also always prints a warning to the user, but in the previous version, it was an optional validation, and it was printed as an error instead of a warning. The results in Table 3 were determined with this validation turned off, so the “Previous Standard Name Errors” category in Table 4 is the number of analyses that would have been in the “Errors” category in Table 3 if the validation had been turned on. Since this validation is technically not new, files that fail the validation would not appear in the count for the “Only New Errors” category, but are counted if we also add in warnings. The reason the count for the “Only New Errors and Warnings” category does not exactly match the count for the “Passing” category in Table 3 is that there are some other smaller issues that account for the difference. For example, in the previous version of the mwtab package, blank Subject IDs in the SUBJECT_SAMPLE_FACTORS would be a validation error, but in the mwTab specification, Subject IDs are optional, so they are no longer errors in the new version of the package. Issues like this affect far fewer datasets, so they were not extensively tabulated and accounted for in Table 4. The majority of datasets that went from Passing to Errors did so due to new validations and making the standard name validation mandatory.

Table 6 shows the number of analyses that failed each validation ID, and Table 7 gives a description of each validation.

The number of analyses that fail each validation varies wildly, from 0 failing to almost all failing. The validations with 0 analyses failing can usually be explained by implication to another validation. For example, take validation B, which is similar to another validation, say validation A, and validation A was added because it was seen in actual datasets, but validation B was added because of its similarity to validation A. Since validation A was confirmed to be an issue, it implies that validation B could also be an issue. For example, “Blank Metabolite(s) in EXTENDED” was added because this issue was found in the METABOLITES and DATA tables, but was not directly found in the EXTENDED table. The validations with much larger numbers of analyses failing are “Standard Column Name Match” and “JSON Schema Error”. “Standard Column Name Match” is explained by there just genuinely being that many datasets with a METABOLITES table that has at least one column with a non-standard name. “JSON Schema Error” has such a large number because it represents many validations under one label. The mwtab package uses the jsonschema package to do some validations, and due to how jsonschema works within mwtab, it is easier to assign one ID to those validations than it is to try to assign an ID to validations within jsonschema. An analysis of the JSON Schema validations showed that the biggest sources of failed validations came from incorrect sub-section values, null-valued sub-section values, and missing required sub-sections, in that order.

Careful observation of Table 6 also reveals some large discrepancies between the number of analyses failing between the mwTab and JSON formats. The top 3, “Duplicate Sub-section”, “Bad Headers”, and “Factor Mismatch” are the easiest to explain. They are issues that by their very nature can only occur in the mwTab format or at least are much less likely to occur in the JSON format. A duplicate sub-section would be when a section, say SUBJECT, has a sub-section repeated, for example, SUBJECT_TYPE. The repetition could have the same information or different information. This is much less likely to occur within the JSON format because sub-sections are keys in a JSON object, and typically, if a key with the same name is added to a JSON object, instead of duplicating, it just overwrites the value for that key. “Bad Headers” occur when there are different numbers of columns and data in rows within a table section. For example, the table in DATA might have 5 columns in the header row, but then a data row might have 7 pieces of data. This does not happen in the JSON format because those tables are stored as lists of JSON objects, and each object always has a 1-to-1 relationship with columns and data. A “Factor Mismatch” occurs when the factors in the SUBJECT_SAMPLE_FACTORS section and the factors in the DATA section are not the same. This cannot occur in the JSON format because it only has the SUBJECT_SAMPLE_FACTORS section; there is no second source of factor information that can be mismatched.

The next discrepancy is “Duplicate Samples in DATA”. This occurs when one or more sample names are used twice as column names in the DATA section. The reason that this does not occur in the JSON format is similar to the explanation already given for the “Duplicate Sub-section” validation. The JSON format does not duplicate the sample keys in its objects used to store the table data; these are instead overwritten so that only the last sample with that name has its data in the JSON format. This means that information is lost when using the downloaded JSON. This also differs from how MW used to construct the JSON in this situation. We analyzed JSON downloads from April 2024 for these datasets, where the key was duplicated in the JSON file. These duplications led to one of the more significant improvements in the mwtab package to allow it to read and write these duplicate keys without losing data. When using the mwtab package, one can read and write datasets that have this duplicate key issue without any problems, and JSON files with this issue that are written out using the mwtab package do not lose data. This is another reason why we do not recommend working with JSON directly downloaded from MW and instead recommend converting mwTab to JSON using the mwtab package.

The next two discrepancies are larger in magnitude, and this time affect the JSON format more than the mwTab format. “Column With No Name” is simply when one of the table sections has a column with no name. Typically, this column is the very last column and is not just a column with no name, but is also a column with all null values. The mwTab format does not have these columns because, during parsing, empty values at the end of lines are removed, so the columns do not end up in the internal representation. The discrepancy in this validation does not translate into a discrepancy in the “Null Column” validation, because so many of these datasets with an extra null column at the end also have other null columns. A “Missing Header” occurs when a table is missing one of its required headers, typically “Metabolite”. This occurs more in the downloaded JSON because, for whatever reason, there are several datasets that have “metabolite_name” instead of “Metabolite”. The mwTab version of the file does not have the issue because, during parsing, the table information is always built using the correct name and does not use whatever is in the file. However, the JSON version is simply read in as is. Both of these discrepancies disappear if using JSON files converted from the mwTab-formatted files using the mwtab package.

Table 8 shows the number of analyses that have a validation issue for each section for each format.

The section with the greatest number of analyses failing is CHROMATOGRAPHY, but this is somewhat misleading because an update to the specification added more required fields to this section after several thousand datasets had already been uploaded without those fields. The second highest on the list is not surprising, since MS_METABOLITE_DATA encompasses both the DATA and METABOLITES tables, and many of the new validations focus on validating the tables in various ways. Within this section, the validations that were failed the most were “Standard Column Name Match”, “Null Column”, and “Possible Bad Column Values”. The two major discrepancies between the formats in this table are in the “no section” category and the METABOLOMICS WORKBENCH section. The reason for the discrepancy in “no section” is that many mwTab-formatted files have a #FACTORS line in them. This is not a valid section name and should not be in any file for any reason. The corresponding JSON versions of the file do not have the empty FACTORS section. The reason for the discrepancy in the METABOLOMICS WORKBENCH section is that, for whatever reason, the PROJECT_ID subsection is blank for many of the mwTab-formatted files, but not for their corresponding JSON versions.

### 3.6. Conversions and Roundtripping

We have mentioned several times that we used the mwtab package to convert the mwTab-formatted files into JSON-formatted files and highlighted how those are often superior to the directly downloaded files, but in our testing of the package, we also performed other conversions. A “roundtrip” in this context refers to reading in a file, writing it back out as one of the 2 formats, and then reading it back in with no loss of information. We performed this round-trip testing from mwTab to mwTab, mwTab to JSON, JSON to mwTab, and JSON to JSON. All conversions were able to be written out and read back in without errors or loss of information, except for 2 files in the mwTab-to-mwTab conversion: AN004560 and AN005763. They both have the same issue, which is a #FACTORS line that should not exist. It causes the NMR_RESULTS_FILE line to be pulled into a FACTORS key that shouldn’t exist, and then, when it is written out, the NMR_RESULTS_FILE line is slightly different and missing the two-letter code and semicolon (NM:) because there is no two-letter code for FACTORS. Not having this two-letter code then causes a parsing error when trying to read in the converted file. It is very difficult to parse in a way to avoid this error without coding for it specifically. We have instead decided to develop a new package, rcha_metab, for which the purpose is to repair, clean, harmonize, and augment (rcha) metabolomics files. This delegates the task of removing problematic lines like the #FACTORS line, among other things, so that the mwtab package does not have to consider those kinds of issues. This package is still in active development and not yet publicly available, but we hope to make it available soon.

## 4. Discussion

The early focus of the mwtab package was to improve programmatic and FAIR access of metabolomics data in MW and to facilitate the creation of clean mwTab-formatted files for deposition into Metabolomics Workbench. After working on the large-scale reuse of MW datasets [19], it was clear that the mwtab package needed to be updated to facilitate this new use case. Mainly, a wider range of validations is needed to identify issues that prevent or hinder large-scale reuse. Also, the new use case spawned our development of another package, rcha_metab, to repair, clean, harmonize, and augment (rcha) the datasets for large-scale reuse [19]. Development of rcha_metab directly fed into the improvements made to version 2.0.0 of mwtab. Also, parsing was improved so fewer files failed to be read in. Error handling was improved so that one “bad” file did not interrupt the batch processing of multiple files. Also, the package is now able to handle duplicate keys, so information is not lost. The validation facilities were greatly expanded and improved. Several validation categories were created, and many more validation tests were added. Now, validation messages are more descriptive, while spurious messages have been reduced. The documentation was updated for all of these changes and can be found at https://moseleybioinformaticslab.github.io/mwtab/ (accessed on 13 December 2025). Automated unit and integration testing were also improved, and over 95% of code lines are now covered. All of these improvements also necessitated improving the mwFileStatusWebsite, which runs the validations discussed here once a week on all downloadable analysis files in MW and then displays the results through its website.

Improvements in parsing in version 2.0.0 of mwtab reduced non-parsability from 162 mwTab-formatted files to 51 mwTab-formatted files, while non-parsability of JSON-formatted files increased from 69 to 124. However, the 55 JSON files that are parsable by the previous version and not the new version are the 48 files with non-dictionary table values and the 7 files that have duplicate keys at the top level, which causes significant issues later in internal representation and validation. Given the improvements in parsability of mwTab-formatted files, the tradeoff is worth it, since the mwTab-formatted files are more reliable for reuse.

With the large addition of validation tests in the 2.0.0 version of mwtab, far fewer analysis files are considered clean (i.e., without issues). Of the files that could be downloaded and parsed, all but 16 mwTab-formatted files and 23 JSON-formatted files had some kind of validation issue, and all but 33 mwTab-formatted files and 44 JSON-formatted files had a serious validation error. This is in stark contrast with the previous version of the mwtab package, where most of the mwTab-formatted files (5497 out of 6117) had no validation issues, and most of the JSON-formatted files (4907 out of 6119) had no validation issues. We also showed a more in-depth breakdown of the largest sources of validation issues in terms of the precise validation IDs and the sections in the files. The validation ID with the most failing analyses was ID 24, JSON Schema Error, and the largest sources of failing analyses within that ID were validations on sub-section values and missing required sub-sections. The top two sections causing analyses to fail validation were CHROMATOGRAPHY and MS_METABOLITE_DATA. Although these evaluations cover a lot, there are still many niche or one-off issues affecting thousands of files. A comprehensive list of everything would not be reasonable to create or maintain, but a list of some of these is provided in the Supplemental Materials.

Based on the evaluations presented here, we have a set of recommendations for MW and the metabolomics field. Actually, all of the recommendations in our last mwtab paper [10] still hold true now. The first major recommendation is to utilize versioning in the analysis files. Both the format requirements and the file contents need to be versioned. While the analysis files include a version key–value pair, it has not been effectively utilized, and all analysis files still list a 1.0 version. Also, the mwTab specification documents for each version should be made available on the MW website. Currently, only the latest version of the mwTab specifications description is available. Without format versioning, it is impossible to apply appropriate validations to analysis files that follow the format requirements at the time of their deposition. We brought the mwtab package up to date with the latest specification version available, but it was impossible to know when exactly some of the changes to the specification occurred since we could not look at the historical changes to the specification. This made it harder to analyze which datasets were failing validation due to new requirements that are unfair to subject them to, since they did not exist at the time of deposition.

The second major recommendation is to standardize/harmonize column names in the METABOLITES table. Without standardization and/or harmonization, the reuse of large numbers of analysis files is pragmatically impossible. As a stopgap, we have developed some tools that are now available in the new version of the mwtab package to help with the standardization/harmonization of column names in the METABOLITES table. The page in our documentation detailing how to use these tools is at https://moseleybioinformaticslab.github.io/mwtab/metadata_column_matching.html (accessed on 13 December 2025). These tools are not specific to metabolite data and could be used for any tabular data, but the prebuilt column finders are all specific to metabolite data and built from the datasets in MW. We believe these tools are timely, given the recent focus on pan-repository metadata harmonization using universal identifiers [20].

The third major recommendation is to adopt UTF-8 character encoding in all mwTab- and JSON-formatted analysis files and in everything returned through MW’s REST interface. Such an adoption will prevent a variety of parsing errors. We also mentioned in our previous paper that a certification system certifying metadata quality and consistency could incentivize better depositions from depositors, and we still believe this to be true, but would recommend focusing on cleaning and improving the data and metadata that is already in MW first. However, such improvements should include a versioning of the contents of the analysis files.

## 5. Conclusions

The version 2.0.0 updates to the mwtab Python package and the MWFileStatusWebsite provide a large number of new validation tests for mwTab- and JSON-formatted analysis files provided by Metabolomics Workbench. The new validation functionality highlights a large number of format, consistency, and value errors in these files provided by the repository. In addition, these updates provide better file parsing and error handling for batch processing and, in total, will facilitate improved curation, FAIRness of the repository, and reuse for large-scale meta-analyses. Based on the work presented here, we have the following main recommendations for Metabolomics Workbench: (1) implement effective format and file versioning of analysis files, (2) standardize and harmonize column (key) names, especially in the METABOLITES table, and (3) adopt UTF-8 character encoding in all mwTab- and JSON-formatted analysis files.

## Figures and Tables

**Table 1:** Summary of changes made to each module in the new 2.0.0 release.

Modules	Changes
cli	Refactored for DRY (Do not Repeat Yourself) purposes. Added error recovery for many commands.
converter	Added error recovery when converting multiple files. ixed write-out errors due to non-ASCII characters.
fileio	Refactored for DRY purposes. dded error recovery when reading and writing multiple files. ixed write-out errors due to non-ASCII characters. ile paths that do not exist will be created instead of raising an error.
mwextract	Added error recovery when operating on multiple files. ile paths that do not exist will be created instead of raising an error.
mwrest	Added error recovery when operating on multiple files.
mwschema	Completely refactored to use the jsonschema package instead of the schema package. dded more validations, particularly on the values of some attributes.
tokenizer	Improved parsing, making some formerly unreadable files readable.
mwtab	Fixed round-trip errors by adding support for duplicate keys. ey/block order is now always the same when writing out. dded convenience properties for attributes in the METABOLOMICS WORKBENCH header. dded convenience methods to convert the tabular sections of the mwTab format to pandas DataFrames and vice versa. mproved file parsing by taking into account some common errors in the mwTab files.
validator	Added many new validations. mproved issue printing by standardizing formatting and greatly reducing the printing of spurious issues. dded a new JSON output with all validation messages tagged with ‘format’, ‘value’, or ‘consistency’.
duplicates_dict	A new module that contains the DuplicatesDict class used to support reading and writing duplicate keys.
metadata_column_matching	A new module for matching metadata columns by names and values. ontains regular expressions, functions, and classes used for matching metadata columns. ontains the “column_finders” dictionary of prebuilt ColumnFinder objects used to match the most common metadata columns in the MW repository.

**Table 2:** List of analysis IDs and the format of files that could not be downloaded through Metabolomics Workbench’s REST API on 22 October 2025.

File Format	Analysis ID
mwTab	AN002312, AN005082, AN005098, AN005099, AN005557, AN006051, AN006148, AN006593
JSON	AN005098, AN005099, AN005557, AN006051, AN006148, AN006593

**Table 3:** The number of analyses that fall into each category for each format when using the previous 1.2.5 version of the mwtab package to validate the analyses.

Issue Category	mwTab	JSON
Passing	5497	4907
Missing	8	6
Parsing Error	162	69
Validation Bug	10	24
Errors	456	1125

**Table 4:** The number of analyses that fall into each category for each format when using the new 2.0.0 version of the mwtab package to validate the analyses.

Issue Category	mwTab	JSON
Passing	16	23
Missing	8	6
Parsing Error	51	124
Validation Bug	0	0
Warnings Only	17	21
Errors	6033	5951
Consistency Errors	538	457
Value Errors	5328	5198
Format Errors	3660	3405
Only New Errors	2634	2548
Only New Errors and Warnings	5717	5640
Previous Standard Name Errors	2926	2883

**Table 5:** Description of each validation issue category is given in Table 3 and Table 4.

Issue Category	Description
Parsing Error	File could not be parsed.
Validation Bug	A coding error was encountered during validation. The file did not necessarily have something wrong with it.
Passing	The file had no validation issues.
Missing	The file could not be downloaded.
Warnings Only	All of the validation issues were only warnings.
Errors	There was at least 1 validation issue with the file that was not a warning.
Consistency Errors	There was at least 1 validation issue due to inconsistencies between data in the file.
Value Errors	There was at least 1 validation issue due to incorrect value(s) in the file.
Format Errors	There was at least 1 validation issue due to the file not meeting the mwTab specification.
Only New Errors	Every issue found is one that has been added in this latest version of the mwtab package.
Only New Errors and Warnings	Every issue found is one that has been added in this latest version of the mwtab package or is a warning.
Previous Standard Name Errors	If the previous version of the mwtab package had validated while using its code for standard name matching, then the file would have errored instead of passing.

**Table 6:** The number of analyses that failed each validation for both downloaded formats.

ID	Validation Short Name	mwTab	JSON
1	Duplicate Sub-section	30	0
2	Bad Headers	44	0
3	Factor Mismatch	27	0
4	Duplicate Sample ID in SSF	6	6
5	Duplicate Factors in SSF	29	28
6	Duplicate Additional Data	346	341
7	Missing Sample ID(s) in SSF	45	75
8	Duplicate Samples in DATA	67	0
9	Metabolite(s) in DATA not METABOLITES	390	363
10	Blank Metabolite(s) in DATA	4	1
11	Duplicate Metabolite(s) in DATA	24	22
12	Metabolite(s) in METABOLITES not DATA	420	371
13	Blank Metabolite(s) in METABOLITES	1	1
14	Duplicate Metabolite(s) in METABOLITES	30	29
15	Standard Column Name Match	3800	3740
16	METABOLITES Bad Standard Values	658	628
17	Missing Implied Column	772	764
18	Paired Columns Value Mismatch	472	457
19	“other_id” Column	1263	1221
20	Multiple Standard Name Match	33	32
21	Missing “sample_id” in EXTENDED	0	0
22	Missing Sample ID(s) in EXTENDED	3	0
23	Bad Metabolite Name	4	0
24	JSON Schema Error	6032	5947
25	Inconsistent Columns	0	0
26	Column With No Name	55	526
27	Null Column	1388	1398
28	Possible Bad Column Values	504	458
29	Duplicate Rows	2	0
30	Duplicate Column Names	18	0
31	Multiple Polarities	11	11
32	No MS or NM Section	2	0
33	Missing METABOLITES Section	0	0
34	Missing Header	54	629
35	Blank Sample ID(s) in EXTENDED	3	0
36	Blank Metabolite(s) in EXTENDED	0	0

**Table 7:** Descriptions of the validations.

ID	Validation Short Name	Description
1	Duplicate Sub-section	The same sub-section appears twice in a section. For example, COLLECTION_SUMMARY shows up twice in COLLECTION. Will only be detected in mwTab-formatted files and not JSON, since duplicate keys tend to overwrite in JSON.
2	Bad Headers	A table section has a mismatch between the number of columns in the data rows and the header row. Can only be found in mwTab-formatted files because tables are lists of dictionaries in JSON, so there is no header line. Each dictionary repeats the column names.
3	Factor Mismatch	The factors in the METABOLITE_DATA section and SUBJECT_SAMPLE_FACOTRS do not match. Can only be found in mwTab-formatted files since the JSON version only has the SUBJECT_SAMPLE_FACTORS section.
4	Duplicate Sample ID in SSF	There are at least two Sample IDs with the same name in the SUBJECT_SAMPLE_FACTORS section.
5	Duplicate Factors in SSF	There are at least two factors with the same name in the SUBJECT_SAMPLE_FACTORS section.
6	Duplicate Additional Data	There are at least two keys in the Additional sample data with the same name in the SUBJECT_SAMPLE_FACTORS section.
7	Missing Sample ID(s) in SSF	There is at least one Sample ID in the METABOLITE_DATA that is not in the SUBJECT_SAMPLE_FACTORS.
8	Duplicate Samples in DATA	There are at least two Sample IDs with the same name in the METABOLITE_DATA section.
9	Metabolite(s) in DATA not METABOLITES	There is at least one Metabolite in the METABOLITE_DATA section that is not in the METABOLITES section.
10	Blank Metabolite(s) in DATA	There is at least one Metabolite in the METABOLITE_DATA section that is blank or null valued.
11	Duplicate Metabolite(s) in DATA	There are at least two Metabolites in the METABOLITE_DATA section with the same name.
12	Metabolite(s) in METABOLITES, not DATA	There is at least one Metabolite in the METABOLITES section that is not in the METABOLITE_DATA section.
13	Blank Metabolite(s) in METABOLITES	There is at least one Metabolite in the METABOLITES section that is blank or null valued.
14	Duplicate Metabolite(s) in METABOLITES	There are at least two Metabolites in the METABOLITES section with the same name.
15	Standard Column Name Match	There is at least one column in the METABOLITES section with a name that matches a standard name.
16	METABOLITES Bad Standard Values	There is at least one column in the METABOLITES section with a name that matches a standard name, and at least one value in that column does not match what values are supposed to look like for that column. For example, a KEGG_ID column has a value that does not look like a KEGG ID.
17	Missing Implied Column	There is at least one column in the METABOLITES section that matches a column that should be paired with another column, but that column is not present. For example, a “retention_index” column should also have a “retention_index_type” column.
18	Paired Columns Value Mismatch	There is at least one column in the METABOLITES section that matches a column that should be paired with another column, but the values in each column do not align. For example, if there is a value in row 5 of a “retention_index” column, then there should also be a value in row 5 for the “retention_index_type” column.
19	“other_id” Column	There is at least one column in the METABOLITES section that matches the column name “other_id”. In general, this column should be avoided and the IDs given a more specific name, such as “kegg_id” or “lab_id”.
20	Multiple Standard Name Match	There is at least one column in the METABOLITES section that matches more than one standard name. Columns like this usually pull double duty and should be separated into multiple columns.
21	Missing “sample_id” in EXTENDED	The EXTENDED_DATA section is missing the required “sample_id” column.
22	Missing Sample ID(s) in EXTENDED	The EXTENDED_DATA section does not have entries for some Sample IDs in the SUBJECT_SAMPLE_FACTORS.
23	Bad Metabolite Name	There is at least one Metabolite name in one of the tables that is close to a name like “metabolite_name” or other names that are likely a mistake and are actually part of a header.
24	JSON Schema Error	One of the many possible validations performed through JSON Schema. Usually, a required subsection is missing or blank, or a value in a subsection does not look right.
25	Inconsistent Columns	One of the tables has at least one row with different columns from another one. Since tables are stored as lists of dictionaries in JSON and the internal MWTabFile object, there is the possibility of a dictionary missing or having extra columns compared to other dictionaries in the same list.
26	Column With No Name	There is at least one column in a table section with no name.
27	Null Column	There is at least one column in a table section where every value is a null value.
28	Possible Bad Column Values	There is at least one column in a table section where 90% of the values are the same, but 10% are different. This could be intentional, but many times it is a mistake.
29	Duplicate Rows	There are at least two rows in a table section that are exactly the same.
30	Duplicate Column Names	There are at least two columns in a table section with the same name.
31	Multiple Polarities	A column in the METABOLITES table was matched to be a “polarity” column, and that column has more than 1 value. It should only ever be positive or negative and not mixed because each analysis is supposed to be one mass spec run. A different mass spec polarity should be in its own analysis.
32	No MS or NM Section	The file does not have an “MS” or “NM” section. At least one or the other is required.
33	Missing METABOLITES Section	The file has a METABOLITE_DATA section but does not have a METABOLITES section.
34	Missing Header	A table is missing a required column, or it has the wrong name. For example, the METABOLITES and METABOLITE_DATA tables require a “Metabolite” column.
35	Blank Sample ID(s) in EXTENDED	The EXTENDED_DATA section has at least one Sample ID that has a blank or null value.
36	Blank Metabolite(s) in EXTENDED	The EXTENDED_DATA section has at least one Metabolite that has a blank or null value.

**Table 8:** The number of analyses that have a validation issue for each section for both downloaded formats, sorted from largest to smallest.

Section	mwTab	JSON
CHROMATOGRAPHY	5679	5621
MS_METABOLITE_DATA	3770	4198
MS	1757	1724
SUBJECT	1730	1695
TREATMENT	1241	1225
no section	895	139
COLLECTION	806	805
SAMPLEPREP	704	695
SUBJECT_SAMPLE_FACTORS	457	471
STUDY	412	411
METABOLOMICS WORKBENCH	303	5
PROJECT	288	287
NM	223	233
NMR_METABOLITE_DATA	122	150
ANALYSIS	82	51
NMR_BINNED_DATA	3	0

## Data Availability

The downloaded files from MW, along with Python scripts for generating the results used in this paper, are available in a Figshare item at https://doi.org/10.6084/m9.figshare.30924581 (posted on 19 December 2025). The 2.0.0 version of the mwFileStatusWebsite is available at https://moseleybioinformaticslab.github.io/mwFileStatusWebsite/ (accessed on 13 December 2025).

## Supplementary Materials

The following supporting information can be downloaded at: https://www.mdpi.com/article/10.3390/metabo16010076/s1. Table S1: List of analysis IDs and the format of files that have parsing errors when trying to read them using the new version of the mwtab package, Table S2: List of analysis IDs and the format of files that have parsing errors when trying to read them using the previous version of the mwtab package, Table S3: List of analysis IDs and the format of files that have validation bugs when trying to validate them using the previous version of the mwtab package, and a list of many small issues found in MW files not mentioned in the paper.

## Abbreviations

The following abbreviations are used in this manuscript:

MW	Metabolomics Workbench
MS	Mass Spectroscopy
NMR	Nuclear Magnetic Resonance
FAIR	Findable Accessible Interoperable Reusable
BSD	Berkeley Software Distribution
REST	Representational State Transfer
API	Application Programming Interface
CLI	Command Line Interface
JSON	JavaScript Object Notation
CSS	Cascading Style Sheets
PYPI	Python Package Index
