# Supplementary material for: A Major Update and Improved Validation Functionality in the mwtab Python Library and the Metabolomics Workbench File Status Website
Source: Metabolites. 2026 Jan 15;16(1):76. doi: 10.3390/metabo16010076 (PMC12844314; doi:10.3390/metabo16010076)
Supplement: Supplementary file 1 [file metabolites-16-00076-s001.zip › metabolites-4083447-supplementary.pdf]

Supplemental Material

Table S1. List of analysis IDs and the format of files that have parsing errors when trying to read them using the version 2.0.0 of the mwtab package.

File Format	Analysis ID
mwTab	AN000258, AN000259, AN000400, AN000401, AN000402, AN000405, AN000598, AN000798, AN000799, AN000800, AN000801, AN000932, AN000933, AN000934, AN000935, AN001552, AN001553, AN001761, AN001762, AN002012, AN002013, AN002094, AN002095, AN002298, AN002343, AN004011, AN004012, AN004013, AN004029, AN004030, AN004031, AN004032, AN004033, AN004034, AN004035, AN004036, AN004037, AN004038, AN004218, AN004270, AN004387, AN004388, AN005475, AN005476, AN005943, AN006100, AN006128, AN006129, AN006142, AN006143, AN006202
JSON	AN000039, AN000074, AN000258, AN000259, AN000400, AN000401, AN000409, AN000427, AN000428, AN000593, AN000602, AN000621, AN000622, AN000623, AN000624, AN000625, AN000703, AN000704, AN000763, AN000764, AN000798, AN000799, AN000800, AN000801, AN000828, AN000831, AN000892, AN000893, AN000932, AN000933, AN000934, AN000935, AN000939, AN001311, AN001373, AN001376, AN001517, AN001761, AN001762, AN001896, AN002009, AN002109, AN002174, AN002343, AN002393, AN002418, AN002533, AN002535, AN002536, AN002549, AN002931, AN003038, AN003270, AN003335, AN003577, AN003592, AN003599, AN003660, AN003798, AN003990, AN003991, AN003992, AN003993, AN003994, AN003995, AN003996, AN004011, AN004012, AN004013, AN004029, AN004030, AN004031, AN004032, AN004033, AN004034, AN004035, AN004036, AN004037, AN004038, AN004201, AN004270, AN004387, AN004388, AN004512, AN004528, AN004699, AN004875, AN005010, AN005011, AN005093, AN005118, AN005166, AN005346, AN005401, AN005409, AN005410, AN005411, AN005412, AN005421, AN005435, AN005517, AN005519, AN005603, AN005605, AN005675, AN005676, AN005717, AN005718, AN005773, AN005943, AN005957, AN005963, AN006019, AN006040, AN006100, AN006128, AN006129, AN006142, AN006143, AN006438, AN007002, AN007003, AN007063, AN007064
Intersection	AN004031, AN000401, AN006143, AN000258, AN004029, AN006142, AN000935, AN005943, AN006100, AN004011, AN004388, AN001762, AN000801, AN004012, AN004032, AN004036, AN004013, AN002343, AN000932, AN004037, AN004035, AN004387, AN004030, AN000934, AN000259, AN006129, AN004270, AN000933, AN006128, AN004034, AN000799, AN004033, AN000400, AN001761, AN000800, AN004038, AN000798

Table S2. List of analysis IDs and the format of files that have parsing errors when trying to read them using version 1.2.5 of the mwtab package.

File Format	Analysis ID
mwTab	AN000402, AN000405, AN000598, AN001296, AN001297, AN001298, AN001299, AN001540, AN001552, AN001553, AN001714, AN001715, AN001712, AN001713, AN001982, AN001988, AN001989, AN001996, AN001997, AN002012, AN002013, AN002035, AN002094, AN002095, AN002105, AN002106, AN002107, AN002108, AN002149, AN002150, AN002192, AN002193, AN002224, AN002225, AN002246, AN002255, AN002256, AN002257, AN002258, AN002259, AN002290, AN002291, AN002292, AN002298, AN002304, AN002305, AN002306, AN002307, AN002313, AN002323, AN002324, AN002325, AN002326, AN002327, AN002328, AN002329, AN002330, AN002331, AN002343, AN002349, AN002355, AN002356, AN002357, AN002358, AN002359, AN002360, AN002363, AN002376, AN002377, AN002378, AN002379, AN002380, AN002381, AN002387, AN002388, AN002396, AN002397, AN002470, AN002471, AN002472, AN002473, AN002525, AN002526, AN002527, AN002528, AN002537, AN002538, AN002539, AN002540, AN002542, AN002543, AN002544, AN002545, AN002670, AN002671, AN002714, AN002715, AN002742, AN002743, AN002744, AN002745, AN002762, AN002763, AN002802, AN002803, AN002804, AN002805, AN002829, AN002830, AN002831, AN002964, AN002965, AN002966, AN002967, AN002970, AN002971, AN002972, AN002973, AN003029, AN003030, AN003139, AN003140, AN003141, AN003142, AN003335, AN003383, AN003384, AN003460, AN003489, AN003490, AN003491, AN003492, AN003493, AN003494, AN003495, AN003496, AN003642, AN003698, AN003699, AN003701, AN003702, AN003703, AN003752, AN003753, AN004095, AN004096, AN004097, AN004098, AN004099, AN004100, AN004218, AN005475, AN005476, AN005482, AN005675, AN005676, AN006100, AN006128, AN006129, AN006142, AN006143, AN006202
JSON	AN000258, AN000259, AN000400, AN000401, AN000798, AN000799, AN000800, AN000801, AN000932, AN000933, AN000934, AN000935, AN000939, AN001311, AN001517, AN001761, AN001762, AN002109, AN002174, AN002393, AN002418, AN002533, AN002535, AN002536, AN003038, AN003270, AN003335, AN004011, AN004012, AN004013, AN004029, AN004030, AN004031, AN004032, AN004033, AN004034, AN004035, AN004036, AN004037, AN004038, AN004270, AN004387, AN004388, AN004875, AN005010, AN005011, AN005093, AN005409, AN005410, AN005411, AN005412, AN005421, AN005675, AN005676, AN005717, AN005718, AN005943, AN005963, AN006040, AN006100, AN006128, AN006129, AN006142, AN006143, AN006438, AN007002, AN007003, AN007063, AN007064
Intersection	AN003335, AN006128, AN006143, AN006142, AN005675, AN006129, AN006100, AN005676

Table S3. List of analysis IDs and the format of files that have validation bugs when trying to validate them using version 1.2.5 of the mwtab package.

File Format	Analysis ID
mwTab	AN000155, AN000156, AN000163, AN000167, AN000171, AN000421, AN000486, AN000646, AN007063, AN007064
JSON	AN000039, AN000421, AN000427, AN000428, AN000486, AN000602, AN000621, AN000622, AN000623, AN000624, AN000625, AN000763, AN000764, AN000788, AN000789, AN000828, AN000831, AN000893, AN001373, AN001376, AN001896, AN001961, AN002549, AN006019

The following is simply an unorganized list of many issues to do with mwTab files usually along with the analysis ID of a file exhibiting that issue:

- Too many elements in the header. There are random filenames/strings mixed in with the key value pairs as well as duplicate keys. AN000037
- Additional "Samples", "Factors", and "metabolite_name" lines that aren't where they are supposed to be or in completely incorrect sections.
- Incorrectly labeled "Samples" sections. For example, a "Samples" line instead having "metabolite_name".
- Slight variations on list headers, such as "Metabolite Name" instead of "metabolite_name". AN000144
- Spacing out of specification. CREATED_ON is biggest offender, but other blocks also have the incorrect spaces according to the specification. AN000086
- Extra tabs at the end of some lines. Mostly on list header lines, "Factors", "Samples", "metabolite_name". AN000152
- Extra spaces in SUBJECT_SAMPLE_FACTORS. AN000152
- Incorrect prefix. For example, MS:INSTRUMENT_NAME instead of CH:INSTRUMENT_NAME in the #CHROMATOGRAPHY section. AN000037
- Unknown section name. For example, FACTORS. AN004560
- RESULTS_FILE in the wrong block. AN002939
- Extra empty key value pairs in JSON tables. There is an extra "":"" at the end of every dictionary. AN000020
- Values in the #METABOLITES block have more values than headers (this could happen in other blocks). Seems to stem from values that have a tab in the value, for example "\t65359". AN005166 AN003577 AN003592 AN003599 AN003660 AN003798 AN004512 AN005118
- Values in the #METABOLITES block have more values than headers (this could happen in other blocks. Similar to the one above, except in this case there are just too many values and not just extra tabs. AN002931 AN002681 AN002682 AN002683 AN002682 AN004201 AN004528 AN004987
- '#' at the beginning of lines that aren't new blocks, for example #MS:MS_RESULTS_FILE. AN004368
- Sub-sections are repeated, such as, “MS:INSTRUMENT_NAME Agilent 6220 TOF“ appearing twice, but not on consecutive lines. Sometimes on consecutive lines. Most are 1 line repeated directly under, and most are in MS section. AN001532 AN001449
- Duplicate factor names in SUBJECT_SAMPLE_FACTORS. AN000379
- Duplicate keys in Additional sample data, but the order matters on the keys. AN001558
- Extra "#END" line at the end of the file. AN001859
- The two-letter code at the beginning of the line is not correct. Example: "_1 CH:CHROMATOGRAPHY" AN002702
- missing the two-letter code, "NM:", at the start of the RESULTS_FILE line. AN002939 AN004560

- Additional data keys in SUBJECT_SAMPLE_FACTORS with too many '='. AN000258 AN000259
- Factor keys in SUBJECT_SAMPLE_FACTORS with too many ':'. AN000400 AN000401
- Newline that shouldn't be there. For example, an email that has a newline just before the @ part. AN000402 AN000598 AN002012 AN002013 AN002094 AN002095 AN004218
- Missing a tab after spaces and before payload. For example, "PR:PHONE   --" has no tab before "--". AN000405
- Every NMR_BINNED_DATA set has malformed JSON without a 'NMR_BINNED_DATA' key and instead just has a 'Data' key. It should be under a 'NMR_BINNED_DATA' key just like all the other data sets. This is a recent change. Older downloaded versions had NMR_BINNED_DATA sections just as expected. AN000041
- Many data sets, around 250, have an extra blank line between all of the lines. AN000001
- EXTENDED sections don't appear in JSON version of the file.
- The full data is copied twice inside the file. Some variations of this have all of the section names copied in twice but with no sub-sections, sort of like a skeleton of a file. The skeletons are sometimes before the actual data and sometimes after. AN005718
- Badly formed results file line and line outside of MS/NMR section. AN001881
